# Coev-web: a web platform designed to simulate and evaluate coevolving positions along a phylogenetic tree

**DOI:** 10.1186/s12859-015-0785-8

**Published:** 2015-11-23

**Authors:** Linda Dib, Xavier Meyer, Panu Artimo, Vassilios Ioannidis, Heinz Stockinger, Nicolas Salamin

**Affiliations:** 1 Department of Ecology and Evolution, University of Lausanne, Lausanne, 1015 Switzerland; 2 SIB Swiss Institute of Bioinformatics, Lausanne, 1015 Switzerland; 3 Computer Science department, University of Geneva, Carouge, 1227 Switzerland; 4 Laboratoire de recherche en neuroimagerie, CHUV, Lausanne, 1011 Switzerland

**Keywords:** Nucleotide, Amino acid, Coevolution, Phylogeny, Simulator, Probabilistic, Web-platform

## Abstract

**Background:**

Available methods to simulate nucleotide or amino acid data typically use Markov models to simulate each position independently. These approaches are not appropriate to assess the performance of combinatorial and probabilistic methods that look for coevolving positions in nucleotide or amino acid sequences.

**Results:**

We have developed a web-based platform that gives a user-friendly access to two phylogenetic-based methods implementing the Coev model: the evaluation of coevolving scores and the simulation of coevolving positions. We have also extended the capabilities of the Coev model to allow for the generalization of the alphabet used in the Markov model, which can now analyse both nucleotide and amino acid data sets. The simulation of coevolving positions is novel and builds upon the developments of the Coev model. It allows user to simulate pairs of dependent nucleotide or amino acid positions.

**Conclusions:**

The main focus of our paper is the new simulation method we present for coevolving positions. The implementation of this method is embedded within the web platform Coev-web that is freely accessible at http://coev.vital-it.ch/, and was tested in most modern web browsers.

## Background

This process of simultaneous evolution has been described in various biological systems and can be an essential process behind changes occurring at the molecular level [1]. Several studies have demonstrated that coevolving sites are critical positions in proteins since they play a role in the folding intermediates [2] and allosteric movements [3–5]. The relevance of these sites has also been shown in disease related protein such as Amyloid beta protein [2]. Moreover coevolving sites play a role in RNA sequences [6, 7] and coevolution is often located on helices that are subject to Watson-Crick constraint (i.e. guanine-cytosine and adenine-thymine complementarity). Several methods have been developed to predict coevolving positions in molecular data [2, 3, 7–10]. However, the full evaluation of the performance of these methods requires large scale simulations and their use is currently impaired by the lack of an appropriate framework to reproduce the process leading to the profiles of coevolution [11]. Indeed, available tools to create in silico nucleotide or amino acid data typically use Markov models to simulate each position independently, which is not appropriate in the case of coevolution [12–15].

We previously developed the Markov model Coev that evaluates the score of coevolution of nucleotide positions using either Maximum Likelihood (ML) or Bayesian inference based on a substitution matrix of size 16×16 [7]. The model describes the transitions between the positions along the branches of a phylogenetic tree and the corresponding profile of coevolution, which represents the set of nucleotides that changed in a coordinated way during sequence evolution.

The Coev model has been developed for nucleotide sequences and is based on a 16 states instantaneous rate matrix *Q* where each state represents a combination of sites. The matrix *Q* contains 4 continuous parameters and a discrete parameter representing the profile *ϕ*. The ratio *d*/*s* indicates the strength of coevolution between a pair of sites. No coevolution is defined when *d*/*s*=1, while larger *d*/*s* ratios represent stronger coevolution. The parameters *r*_1_ and *r*_2_ represent the rate of single substitutions for position 1 and position 2, respectively, and they can take arbitrary values when the pair is highly coevolving but will be more accurately estimated when the pair is not coevolving. To assess the coevolution between two sites, we can also calculate a *Δ*AIC score to compare the likelihood of the the Coev model with the likelihood of an independent model of evolution [16].

(1) $${}  Qij = \left\{ \begin{array}{ll} 0&\text{if \textit{i} and \textit{j} differ by two nucleotide positions},\\ s &\text{if \textit{i}} \in \phi \,\text{and \textit{j}} \not\in \phi, \\ d &\text{if \textit{i}} \not\in \phi \,\text{and \textit{j}} \in \phi,\\ r_{1}&\text{if \{\textit{i}, \textit{j}\}} \notin \phi \,\text{and if \textit{i} differs from \textit{j} at position 1}, \\ r_{2}&\text{if \{\textit{i}, \textit{j}\}} \notin \phi \,\text{and if \textit{i} differs from \textit{j} at position 2} \\ \end{array} \right.  $$

The likelihood of the Coev model also depends on the profile of coevolution for the pair of sites. The total number of profiles depends on the alphabet and it equals to 192 in the case of a nucleotide alphabet (size 4). The Coev model estimates the probability of a pair of positions *X* coevolving along a phylogenetic tree with topology *τ* and branch lengths *ν* as described by *P* *r* *o* *b*(*X*|*ϕ*,*s, d,* *r*_1_,*r*_2_,*τ*,*ν*).

For simplicity, we assume that *τ* and *ν* are known and are not estimated [7]. We use Felsenstein’s pruning algorithm [17] to evaluate the likelihood of the model. This is done by calculating, for each branch of a phylogenetic tree, the transition probability matrix *P*(*t*)=*e* ^*Qt*^, where the branch length *t* is a finite time interval. Since the matrices size, *n* ^4^, grows exponentially with the size of the alphabet, the matrix exponentiation requires high performance computing. We therefore implemented the software in C/C++ and used several external tools for matrix exponentiation (Linear Algebra PACKage) and optimisation (nlopt, library for nonlinear optimisation; [7, 18]). These dependencies might be difficult to install for non-expert users. For this reason, we designed a user friendly and publicly available web server to analyse and simulate coevolution in nucleotide sequence data.

In this Software paper, we present two novel extensions of Coev model, that enables the simulation of coevolving pairs of nucleotide or amino acid along a phylogenetic tree. The software is accessible through a web platform, hosted on a high performance computing infrastructure (http://www.vital-it.ch). The user friendly Coev-web platform also allows the user to evaluate the probability of coevolving nucleotide and their respective evolutionary profile based on the aligned sequences and a phylogenetic tree.

## Implementation

### Coev-web platform workflow

**Fig. 1:**
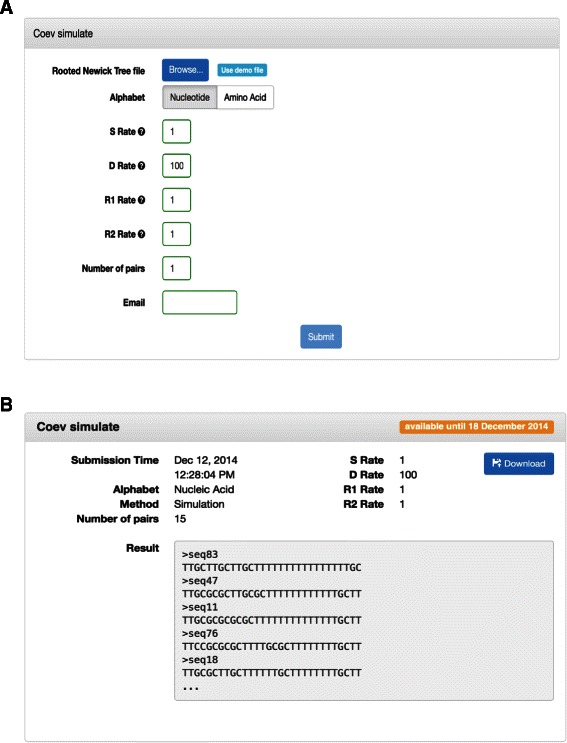
Simulation interface of the Coev-web platform. Part **a** shows the job submission page while part **b** gives an example of the results obtained for 15 pairs

Through the Coev web-interface, available on Vital-it, as illustrated in Fig. 1, the user can: (1) simulate a pair of coevolving positions along a fixed phylogenetic tree using *s*, *d*, *r*_1_ and *r*_2_ parameters (2) estimate the coevolving score and *s*, *d*, *r*_1_ and *r*_2_ parameters using maximum likelihood or Bayesian framework within DNA sequences.

Different requirements are necessary for each type of experiment as detailed in the Usage paragraph. When the user submits the form, several controls are performed to verify if the form is complete and correctly filled. If this is not the case, an error message is displayed to inform the user about the issue.

When the process is completed, the user receives an e-mail containing the results. For the simulation step, it will be composed of the alignment file with the simulated sequences in FASTA format. For the evaluation step under ML, it will contain the values of the rate parameters that were optimised and the best profile. A *Δ*AIC associated value is also provided to the users as a testing criterion that reflects how coevolving a pair is. The bigger the value is the more reliable the results are [16]. Whereas for the Bayesian evaluation, it will contain a log file readable by the graphical tool for visualisation and diagnostics of MCMC output Tracer [19].

The time to complete the evaluation or simulation runs depends on the size of the phylogenetic tree and other parameters such as the number of iterations, the sampling frequency, etc.

### Usage

Different inputs from the users are necessary for each method available in the web-platform. First, the simulation of pairs of coevolving positions requires the upload of a tree file in Newick format, the specification of the 4 continuous parameters of the Coev model and the number of pairs to simulate. The user will need to take the following steps on the web-interface:

1. Upload the rooted binary phylogenetic tree in Newick format
2. Specify the values of the 4 continuous evolutionary rates (*s*, *d*, *r*_1_ and *r*_2_)
3. Set the number of pairs to simulate under the same coevolving profile
4. Provide an e-mail address

Second, the evaluation of the score of coevolution of a pair of positions requires the upload of a multiple sequence alignment (in standard FASTA format) and the corresponding phylogenetic tree (in standard Newick format) before specifying the two positions along the sequence that should be analysed. Finally, the user will have to select the type of inference to use (either ML or Bayesian). The user will thus take the following steps on the web-interface:

1. Upload the aligned sequences in FASTA format
2. Upload the rooted binary phylogenetic tree in Newick format
3. Specify the inference method: ML or Bayesian
4. Set the positions that will be tested using two input fields
5. Provide an e-mail address

For the Bayesian inference, there are some extra parameters to fill: the number of iterations, the burn-in and the sample frequency. To make things simple, we could consider the Bayesian algorithms as iterative algorithms that repeat themselves several times by changing the model parameters values. The number of times they iterate is defined by the “iterations” value, when the “burn-in” is a term that describes the practice of throwing away the initial iterations before the chain reached the equilibrium representing the posterior distribution. The sampling frequency is the frequency of the algorithm reporting. For example, when the sampling is set to 1,000, the software reports its state every 1,000 iterations. By default we advise the Coev-web platform users to consider 1,000,000 iterations and a burn-in of 1,000 and a sampling frequency of 1,000 for the Bayesian implementation.

### Data curation

During the analysis that evaluates the score of coevolution, we took particular care to check the input file containing the alignment. Since the model cannot consider gaps sites and fully conserved sites, we therefore filter the alignment by removing conserved sites and sites containing gaps. We also remove all sites containing letter that do not belong to the nucleic alphabet {A, C, G, T}. Once processed, the alignment file can be downloaded by the user to validate the filtering.

## Results and discussion

We developed a new and user-friendly web platform, called Coev-web, that provides an easy access to the model described in [7]. We discuss below new extensions to the existing Coev model that enable more generality in the type of data being analysed and propose the first tool to simulate coevolving positions.

***Extension1: model generalisation***

The original Coev model was created to analyse nucleotide sequences and involved the search through the 192 profiles existing for a nucleotide alphabet [7]. The Coev-web platform provides a user friendly interface to evaluate the score of coevolution using either ML or Bayesian frameworks. We extended the capabilities of the Coev model by increasing the alphabet size of the substitution matrix from *n*=4 to *n*=20 to analyse amino acid sequences. This resulted in a drastic increase of the computational complexity of the analyses. Although the 4 continuous parameters *s*, *d*, *r*_1_ and *r*_2_ apply to both data types, the size of the instantaneous rate matrix increases from 16×16 to 400×400, which makes the matrix exponentiation steps required to calculate the likelihood much more computationally demanding. The number of possible profiles also increases drastically since for an alphabet of size *n*, it amounts to $\sum _{k=2}^{n} \left (\frac {n!}{(n-k)!} \times \frac {1}{k!} \right)^{2}$. For amino acids, the number of profiles to search through is increasing to an order of 10^21^ possible profiles.

The increased complexity of the computations to generalize the Coev model to amino acids requires a high performance computing approach. We therefore implemented the software in C/C++ and used several external tools to speed up the costly matrix exponentiation [7, 20]. The dependencies might be difficult to install for non-expert users. For this reason, we designed the publicly available Coev-web platform that analyses coevolving pairs of positions for nucleotide and amino acid sequences.

**Fig. 2:**
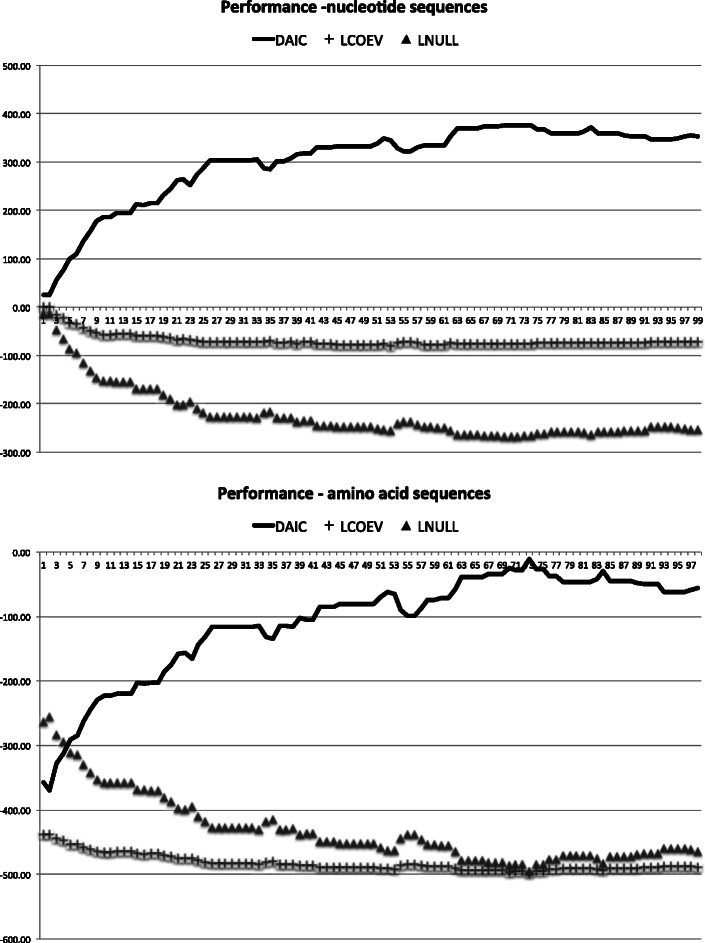
Correlation between the number of lineages with double substitutions and the likelihood difference between Coev model and independent model for amino acid and nucleotide sequences. The X axis reflects the number of lineages with double substitutions. In both plots the same tree is used and it is composed of 100 leaves. The likelihood difference increases as X increases. The likelihood difference represented by *Δ*AIC shows that the Coev model is preferred to the independent model for amino acid and nucleotide sequences especially when X is big. (1.) The combinations used for nucleotide experiment are Adenine-Adenine (AA) and Thymine-Thymine (TT). (2.) The combinations used for the amino acid experiment are Alanine-Alanine (AA) and Threonine-Threonine (TT)

We illustrate the use of the evaluation tool on protein sequences by calculating the correlation between the number of lineages with double substitutions and the likelihood difference between Coev model and independent model for amino acid and nucleotide sequences. To check whether our new method can distinguish coevolving from co-inherited pairs of positions using amino acid model as described by [6], we designed an experiment with a tree composed of two sub-trees of 50 leaves. The branch lengths of the tree were randomly generated from a normal distribution with mean = 0.5. We assigned Alanine-Alanine (AA) combination to the leaves of the first subtree and Threonine-Threonine (TT) combination to the leaves of the second subtree. Then we exchanged combinations between first and second sub-trees successively 100 times. Each time we exchanged two combinations, we evaluated the likelihood difference between the Coev model and the independent model using ML implementation and the number of co-substitution. The likelihood difference represented by *Δ*AIC shows that the Coev model is preferred to the independent model for amino acid especially the number of double substitution is big (Fig. 2). This experiment validates Dib et al. ([7]) assumption using amino acid alphabet and suggests that Coev model can distinguish coevolving from co-inherited pairs.

***Extension2: Simulating coevolving pairs***

**Fig. 3:**
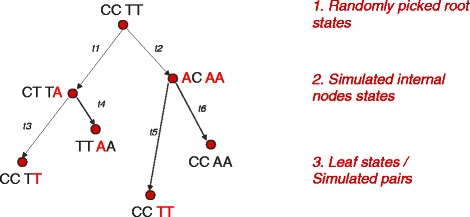
Simulation. We present the simulation steps of two nucleotides pairs along a phylogenetic tree of 4 leafs. In red we highlight the nucleotide changes. (1.) We randomly pick a state at the root. (2.) We assign internal node states using the transition probability matrix *P*(*t*)=*e* ^*Qt*^ where *Q* is the Coev instantaneous rate matrix and t is the branch length [7]. (3.) The simulated pairs are the pairs assigned to the leafs of the phylogenetic tree

The Coev-web platform further offers another novelty by allowing the simulation of dependent nucleotide pairs of position (Fig. 1). This is an important tool that was so far missing to evaluate the performance of methods to analyse coevolution [11]. Given a tree in Newick format and the values of the 4 continuous parameters of the Coev model, the software randomly picks a coevolving profile, a state at the root of the tree and lets this state evolve along the branches of the tree according to the Coev substitution matrix (Fig. 3). Because of the Markovian properties of the Coev model, the waiting time for a substitution to occur along a branch of the phylogenetic tree is exponentially distributed. When a substitution does occur, the arrival state is drawn from a frequency vector evaluated from the matrix *Q*. The software therefore simulates pairs of positions along each branch of a tree by assigning a state composed of two letters from the given alphabet to the leaves.

**Fig. 4:**
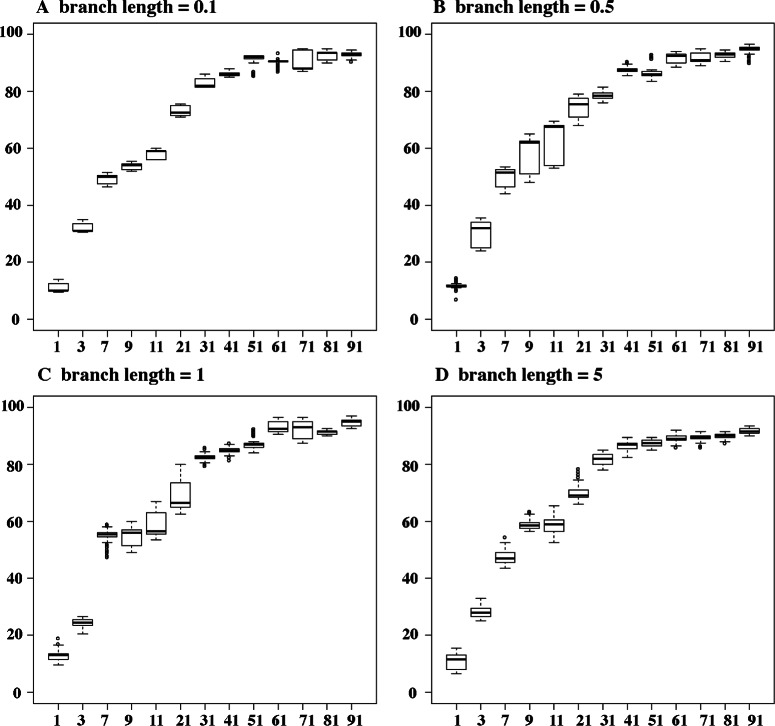
Simulation plots. We plot the proportion of combinations simulated, that belong to the profile {AA, CC}, against *d*/*s* ratio along different branch lengths (**a** for 0.1; **b** for 0.5; **c** for 1; **d** for 5). Each box plot is obtained by varying the *r*_1_ and *r*_2_ rates within the range [1,100] and by randomly picking an ancestral state from the frequency vector issued from the matrix *Q*

We illustrate the use of our simulation tool by evolving the profile of coevolution {AA,CC} along a branch of different lengths. We varied the continuous rate parameters (*s*, *d*, *r*_1_, *r*_2_) and observed that the proportion of coevolving combinations becomes higher when *d* is larger than *s* (Fig. 4). This observation is true regardless of the branch lengths tested. We are therefore able to simulate coevolving and non-coevolving sites by simply changing the values of the *s* and *d* parameters.

## Conclusions

Coev-web is the first web platform that gives access to a phylogenetic-based simulator of nucleotide or amino acid coevolving positions. It also provides a way to evaluate the score of coevolution between pairs of positions in a nucleotide or amino acid sequence that can predict coevolving positions and their evolutionary profile based on the aligned sequences and a phylogenetic tree.
